# Interannual Variations in Terrestrial Net Ecosystem Productivity and Climate Attribution in the Southern Hilly Region of China

**DOI:** 10.3390/plants13020246

**Published:** 2024-01-15

**Authors:** Xin Qi, Shuhua Liu, Shaoan Wu, Jian Wang, Jiaming Wang, Chao Zheng, Yong Wang, Yang Liu, Quan Luo, Qianglong Li, Liang Wang, Jie Zhao

**Affiliations:** 1 Changsha Natural Resources Comprehensive Survey, China Geological Survey, Changsha 410600, China; qixin920613@gmail.com (X.Q.); wushaoanbest@163.com (S.W.); wangjian11020401@163.com (J.W.); cszxzc77777@163.com (C.Z.); wyong199012@163.com (Y.W.); liqianglongjtt@163.com (Q.L.); 2 Shandong Provincial Key Laboratory of Water and Soil Conservation and Environmental Protection, College of Resources and Environment, Linyi University, Linyi 276000, China; liushuhua@lyu.edu.cn (S.L.); wangliang.cn@163.com (L.W.); 3 College of Natural Resources and Environment, Northwest A & F University, Yangling 712100, China; wangjiaming@nwafu.edu.cn

**Keywords:** net ecosystem productivity, climate attribution, southern hilly region of China, nocturnal warming

## Abstract

The vegetation ecosystem in the southern hilly region of China (SHRC) plays a crucial role in the country’s carbon reservoir. Clarifying the dynamics of net primary productivity (NPP) in this area and its response to climate factors in the context of climate change is important for national forest ecology, management, and carbon neutrality efforts. This study, based on remote sensing and meteorological data spanning the period 2001 to 2021, aims to unveil the spatiotemporal patterns of vegetation productivity and climate factors in the southern hilly region, explore interannual variation characteristics of vegetation productivity with altitude, and investigate the response characteristics of NPP to various climate factors. The results indicate that from 2001 to 2021, the annual average NPP in the southern hilly region had a significant increasing trend of 2.13 ± 0.78 g m^−2^ a^−1^. The trend of NPP varies significantly with altitude. Despite a general substantial upward trend in vegetation NPP, regions at lower elevations exhibit a faster rate of increase, suggesting a diminishing difference in the NPP of different elevation ranges. The overall rise in average temperature has positive implications for the southern hilly region, while the impact of precipitation on vegetation NPP demonstrates noticeable spatial heterogeneity. Regions in which vegetation NPP is significantly negatively correlated with precipitation are mainly concentrated in the southern areas of Guangdong, Fujian, and Jiangxi provinces. In contrast, other regions further away from the southeastern coast tend to exhibit a positive correlation. Over the past two decades, there has been an asymmetry in the diurnal temperature variation in the SHRC, with the nighttime warming rate being 1.8 times that of the daytime warming rate. The positive impact of daytime warming on NPP of vegetation is more pronounced than the impact of nighttime temperature changes. Understanding the spatiotemporal patterns of NPP in the SHRC and the characteristics of its response to climate factors contributes to enhancing our ability to protect and manage vegetation resources amidst the challenges of global climate change.

## 1. Introduction

As a pivotal component of the global terrestrial ecosystem, land surface vegetation plays a crucial role in energy transfer, the water balance, the carbon cycle, and climate regulation [1,2]. One vital indicator of ecosystem function is net primary productivity (NPP), defined as the net carbon uptake by plants through photosynthesis (the difference between the carbon assimilated during photosynthesis and that released during plant respiration) [3,4]. NPP is extensively employed for measuring vegetation dynamics and assessing ecological security [5,6]. Recently, it has become possible to expand NPP studies from the traditional site scale to regional and global scales [7,8,9] by using remote sensing techniques which now offer convenient and efficient means for NPP estimation. Changes in vegetation NPP in the southern hilly region of China (SHRC), the area of China with the most concentrated distribution of hills, have important implications for China’s carbon sink, but the region has not been the subject of any research specifically focused on this topic. However, previous studies have analyzed the interannual variations in NPP of many different vegetation ecosystems at regional or national scales. For example, Du et al. [4] employed the Carnegie–Ames–Stanford Approach (CASA) model to invert and analyze the spatiotemporal patterns of the interannual variations in NPP of forest ecosystems in China from 1982 to 2015, while Teng et al. [6] employed the CASA model and the residual trend method to estimate the actual NPP, potential NPP, and anthropogenic NPP, along with their variations, in the Qilian Mountains in Northwest China from 2001 to 2012. The spatiotemporal variations in the NPP of temperate grasslands in China over the past two decades were analyzed Ma et al. [5] using remote sensing data. Recent studies have shown significant variations in the impact of climate change on vegetation dynamics at different altitudes [10]. However, it is still unclear whether there are distinct interannual variations in vegetation productivity along the altitudinal gradient in the SHRC.

The NPP of plant ecosystems is influenced by various environmental factors, but climate factors have been shown to be the primary determinants of their carbon sequestration capacity [4,10,11,12]. For instance, Peng et al. [11] suggested that recent climate changes have led to an increase in NPP in the northern forests of central Canada. Wang et al. [12] analyzed the impact of temperature and precipitation on the NPP in China at the beginning of the 21st century and found a relatively weak correlation between NPP and annual average temperature [12]. In the northern and central regions of China, NPP is significantly positively correlated with the annual total precipitation, whereas in the eastern Tibetan Plateau and Sichuan Basin, there is a negative correlation [12]. Du et al. [4], relying on meteorological and remote sensing data, observed that elevated temperatures and solar radiation have raised the annual NPP of forests in China, while decreased precipitation has diminished the annual NPP. Despite these prior studies analyzing the response of vegetation productivity to climate factors in specific regions, vegetation types and time periods, there has been relatively little attention given to the response of vegetation productivity to climate change in the SHRC.

Over the past half-century, there has been an asymmetry in the warming trend between daytime and nighttime, with nighttime warming rates surpassing those during the daytime [13,14]. This asymmetry in diurnal temperature variation is influenced by various factors such as deforestation [15,16], land-use and land-cover changes [17,18], atmospheric aerosols [19], precipitation [20,21], cloud cover [21,22], solar radiation [23,24], and planetary boundary layer height [13]. For instance, an increase in total cloud cover tends to reduce the amplitude of daytime warming while enhancing the rate of nighttime warming [22], because the reduction in shortwave solar radiation reaching the Earth’s surface during the day is offset by an increase in the downward longwave radiation received by the surface at night [22]. The asymmetry in the rates of daytime and nighttime warming has also gained attention due to its impact on vegetation activity [25]. Research findings based on satellite observations and field experiments indicate differences in the response of vegetation dynamics and NPP to daytime and nighttime warming [25,26,27,28]. For example, Peng et al. [26] found that in the majority of humid and cold ecosystems in northern regions, daytime warming typically exhibited a positive impact on the increase in vegetation greenness, whereas nighttime warming was usually negatively correlated with vegetation greenness. However, these studies have primarily been conducted in high latitudes, high altitudes, or inland arid regions. Research on the response of vegetation in the SHRC to daytime and nighttime warming remains scarce.

Based on remote sensing data and meteorological data from 2001 to 2021, this study attempts to: (1) reveal the spatiotemporal patterns of vegetation NPP and climate factors (i.e., average temperature, daytime temperature, nighttime temperature, and precipitation) in the SHRC; (2) explore the differences in the interannual variation characteristics of vegetation NPP with altitude; (3) investigate the response characteristics of NPP to different climate factors (i.e., average temperature, daytime temperature, nighttime temperature, and precipitation). The aim of the above analysis is to provide reference values for the sustainable ecology and management of vegetation in the SHRC.

## 2. Data and Method

### 2.1. Dataset

The vegetation NPP data utilized in this study were acquired from the Global NPP dataset version 6.0 (MOD17A3HGF 6.0) of the National Aeronautics and Space Administration (NASA) Earth Observing System (EOS)/Moderate Resolution Imaging Spectroradiometer (MODIS) and cover the years 2001 to 2021. The data were obtained from https://lpdaac.usgs.gov/ (accessed on 12 November 2023) and have a temporal resolution of 1 year, spatial resolution of 500 m, and are formatted in HDF [29]. The MODIS Reprojection Tool (MRT) was employed to batch stitch, format conversion, and reproject the original NPP images. After batch stitching, standardizing coordinates, and cropping, vegetation NPP data for the SHRC for the years 2001 to 2021 were generated. Each year’s NPP data were multiplied by a conversion factor, and invalid values were excluded from the analysis and calculations. The MOD17A3HGF version 6.0 dataset represents an improved product which is based on the BIOME-BGC model and the light use efficiency model [5,30]. It utilizes a new biome properties look-up table (BPLUT) and daily meteorological data from the Global Modeling and Assimilation Office (GMAO) to simulate NPP values, thereby enhancing the accuracy of NPP estimation [31]. This dataset, which has been validated and utilized in diverse global research studies [32,33,34], was employed here to resample NPP data using the mean method with a resolution of 0.05 degrees.

Historical monthly-average daily maximum and minimum temperatures (T_max_ and T_min_) and precipitation data spanning from 2001 to 2021 were acquired from the WorldClim historical monthly weather dataset. This dataset, derived from CRU-TS-4.06 by the Climatic Research Unit at the University of East Anglia, has been extensively employed in research exploring the relationship between regional vegetation NPP and climate change. It has a spatial resolution of 0.04° × 0.04° and employs angular-distance weighting interpolation. The data were downsized and bias-corrected using WorldClim 2.1 [35]. Here, based on previous studies [26,27], we use T_max_ and T_min_ to represent daytime temperature and nighttime temperature, respectively, while T_mean_ is computed as the average of T_max_ and T_min_. To align with the resolution of the vegetation NPP data, the historical monthly climate datasets were further aggregated to 0.05° × 0.05° using the nearest neighbor method.

**Figure 1:**
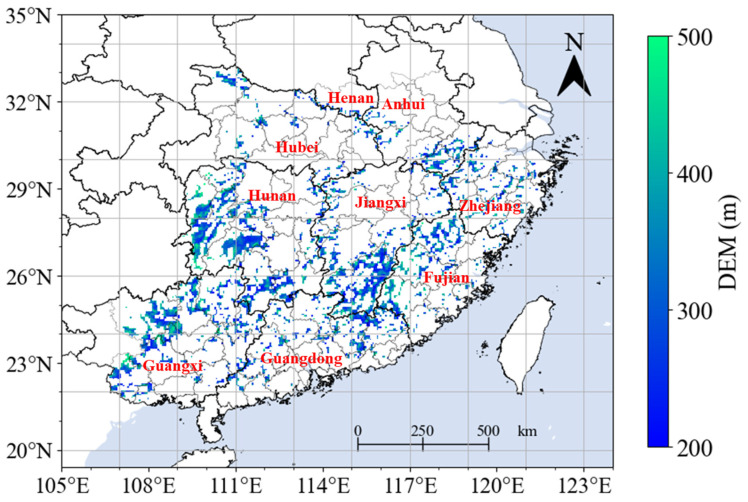
Spatial distribution of elevation (DEM) in southern hilly region of China.

The spatial distribution of elevation at a 0.0083° spatial resolution for the SHRC is based on NASA Shuttle Radar Topographic Mission (SRTM) Digital Elevation Model (DEM) data. This SRTM dataset, renowned for its high accuracy and open accessibility, has been widely utilized in environmental analyses [36,37,38]. The dataset was acquired from the Research Center for Environmental and Data Sciences at the Chinese Academy of Sciences (https://www.resdc.cn/data.aspx?DATAID=123, accessed on 20 December 2023) and resampled to a spatial resolution of 0.05° using the mean method. In this study, hilly areas were designated as regions with elevations ranging from 200 to 500 m (Figure 1). Altitudinal levels in the study area were classified into three groups: low-altitude (200 m < DEM ≤ 300 m), mid-altitude (300 m < DEM ≤ 400 m), and high-altitude (400 m < DEM ≤ 500 m).

### 2.2. Method

#### 2.2.1. Linear Trend Analysis

We used least squares linear regression for the trend analysis of climate variables and NPP. Positive and negative regression coefficients were interpreted as indicating increasing or decreasing trends, respectively [28,39]. The significance level of the linear regression coefficients was evaluated with a two-tailed *t*-test [27]. Additionally, pixel-based calculations were employed to analyze the spatiotemporal patterns of trends in climate variables and NPP in vegetated areas in the SHRC during 2001–2021.

#### 2.2.2. Partial Correlation Analysis

We used a first-order partial correlation analysis to examine the impact of temperature and precipitation on the NPP of vegetation in the SHRC from 2001 to 2021. The first-order partial correlation coefficient can be calculated from the correlation coefficient by using the following formula [40]:

(1) $$r_{xy\cdot z}=\frac{r_{xy}-r_{xz}r_{yz}}{\sqrt{1-r_{xz}^{2}}\sqrt{1-r_{yz}^{2}}}$$

where *r_xy__⋅z_* is the first-order partial correlation coefficient between *x* and *y* after removing the influence of variable *z*. *r_xy_*, *r_xz_*, and *r_yz_* are the correlation coefficients between the two variables.

The formula for calculating the correlation coefficient is [40]:

(2) $$r_{xy}=\frac{\sum_{i=1}^{n}\left(x_{i}-\bar{x}\right)\left(y_{i}-\bar{y}\right)}{\sqrt{\sum_{i=1}^{n}{\left(x_{i}-\bar{x}\right)}^{2}\sum_{i=1}^{n}{\left(y_{i}-\bar{y}\right)}^{2}}}$$

where *r_xy_* is the correlation coefficient between variables *x* and *y*. *x_i_* and *y_i_* are individual data points for *x* and *y*. *x* and *y* are the means of *x* and *y* respectively.

The significance of the partial correlation coefficient is conducted using a *t*-test, given by:

(3) $$t=\frac{r_{xy,z}}{\sqrt{1-r_{xy,z}}}\sqrt{n-m-1}$$

where *n* represents the sample size, and *m* represents the number of independent variables.

In addition, we employed a second-order partial correlation analysis when examining the impact of daytime and nighttime temperatures on the NPP of vegetation. Specifically, the partial correlation coefficient between NPP and T_max_ was calculated by restricting T_min_ and the precipitation and, similarly, the partial correlation coefficient between NPP and T_min_ was determined by constraining T_max_ and precipitation. The formula for calculating the second-order partial correlation coefficient is [40]:

(4) $$r_{xy\cdot z1z2}=\frac{r_{xy\cdot z1}-r_{xz2\cdot z1}r_{yz2\cdot z1}}{\sqrt{1-r_{xz2\cdot z1}^{2}}\sqrt{1-r_{yz2\cdot z1}^{2}}}$$

where *r_xy__⋅z_*_1*z*2_ is the first-order partial correlation coefficient between *x* and *y* after removing the influence of variables *z*1 and *z*2. *r_xy·z_*_1_, *r_xz_*_2*·z*1_ and *r_yz_*_2*·z*1_ are the first-order partial correlation coefficients between the two variables.

## 3. Results

### 3.1. Spatial–Temporal Variation of Climatic Factors

**Figure 2:**
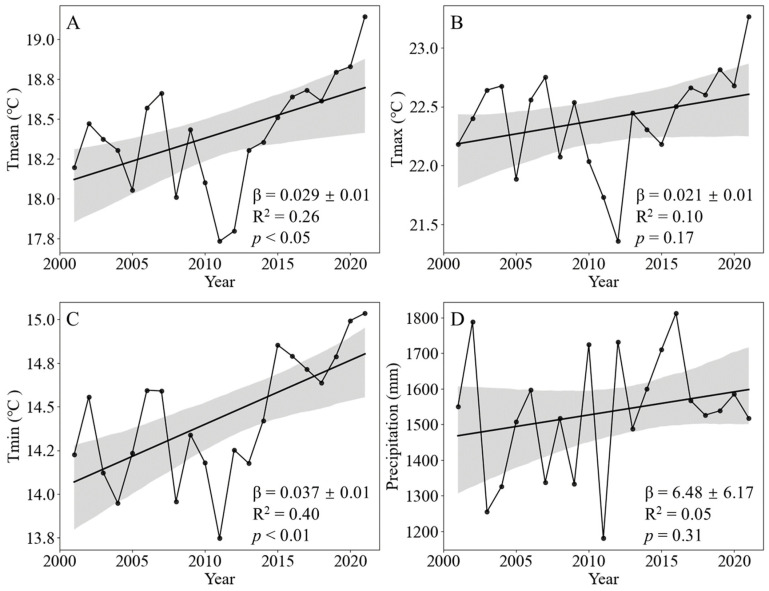
Variations of annual climate factors in the southern hilly region of China during 2001–2021. (**A**) Average temperature (T_mean_). (**B**) Daily maximum temperature (T_max_). (**C**) Daily minimum temperature (T_min_). (**D**) Total annual precipitation. Shading denotes 95% prediction intervals.

The average annual temperature (T_mean_) in the SHRC increased significantly at a rate of 0.29 °C/decade (*p* < 0.05) on average from 2001 to 2021 (Figure 2A). The linear fitting equations reveal that T_min_ has increased by 0.37 °C/decade (*p* < 0.01), while T_max_ has increased by 0.21 °C/decade (*p* > 0.05), meaning the nighttime warming rate was approximately 1.8 times higher than the daytime warming rate (Figure 2B,C). This observation suggests an asymmetry in the warming patterns between the daytime and nighttime over the past two decades (Figure 2B,C). For precipitation, there is no significant interannual variation (*p* > 0.05) in the SHRC at the regional scale for the period from 2001 to 2021 (Figure 2D).

**Figure 3:**
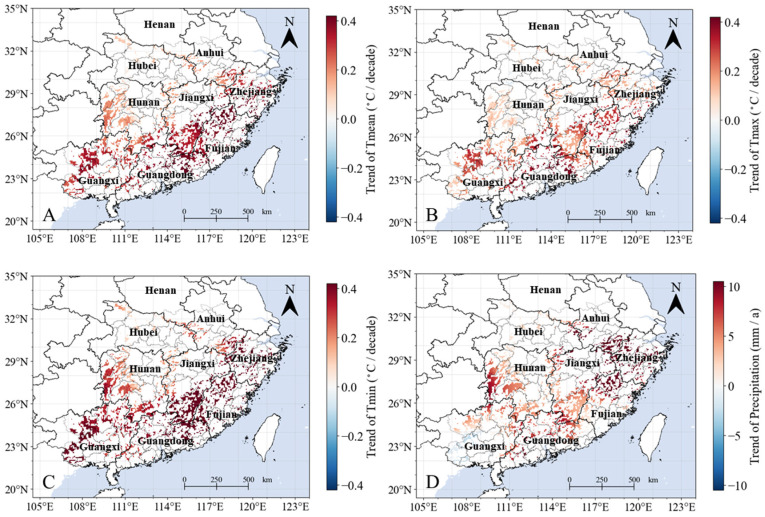
Spatial distribution of linear trends in climate factors from 2001–2021. (**A**) Mean daily temperature (T_mean_). (**B**) Daily maximum temperature (T_max_). (**C**) Daily minimum temperature (T_min_). (**D**) Total annual precipitation (mm). The letter “a” represents the year.

Spatially, the increase of T_mean_ was extensive, being observed across more than 99.5% of the study area, with 59.7% of the pixels showing significant increases (*p* < 0.05) (Figure 3A). Across the entire study area, the southern regions of Guangxi, Guangdong, Fujian, and Jiangxi provinces experienced the highest rates of temperature increase, while the provinces situated further north exhibited lower rates of increases in T_mean_ (Figure 3A). It is noteworthy that only 9.5% of T_max_ shows a significant increasing trend, whereas the proportion of T_min_ exhibiting a significant increase reaches 78.7% (Figure 3B,C). This result indicates a pronounced asymmetry in diurnal temperature changes in the study area, with the majority of the rise in T_mean_ from 1982 to 2015 being driven by an increase in nighttime temperatures. Similarly to the spatial pattern of T_mean_, areas with a significant increase in both T_max_ and T_min_ are predominantly located in lower-latitude regions (Figure 3B,C). Additionally, during the same period, there is no significant decreasing trend in T_mean_, T_max_, and T_min_ in the study area. For precipitation, although 92.7% of the trends show an upward trajectory, these are only statistically significant (*p* < 0.05) for 4.8% of the region, primarily in areas of the southeastern part of Anhui Province and the northwestern part of Fujian Province (Figure 3D). No significant downward trend in precipitation was found anywhere in the study area over the same period (Figure 3D).

### 3.2. Spatial–Temporal Variation of NPP

**Figure 4:**
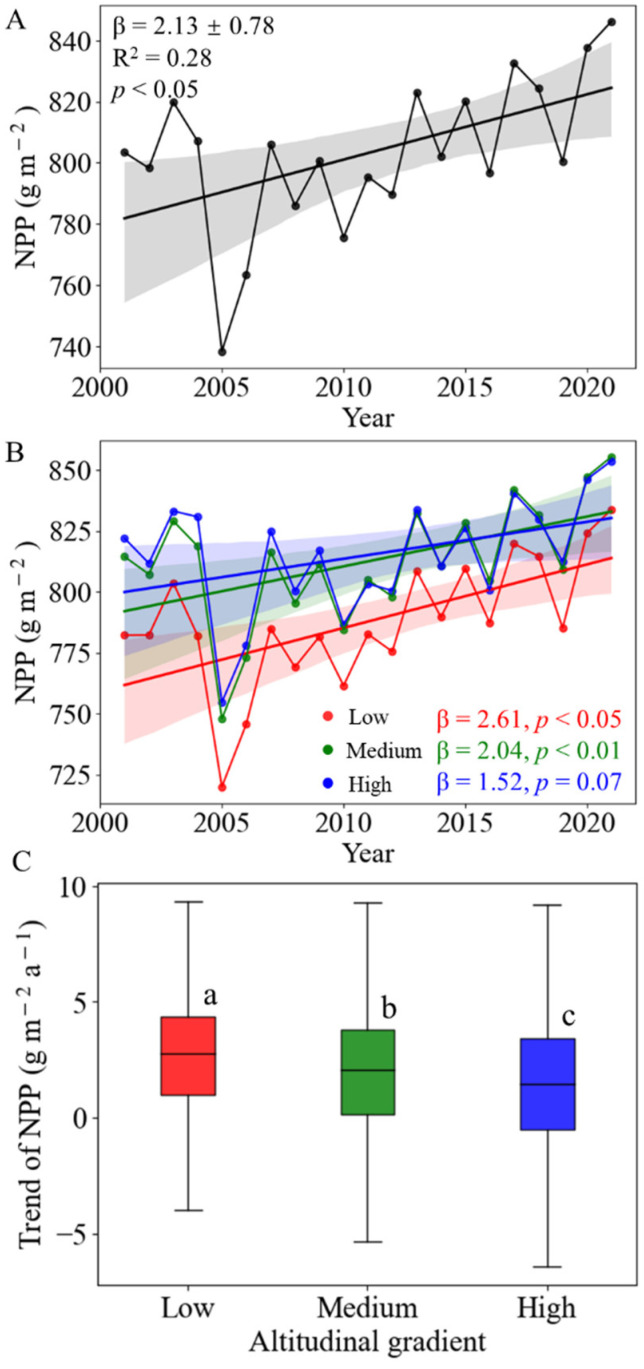
Variations of annual NPP in the southern hilly region of China (SHRC) from 2001–2021. (**A**) Annual NPP and trend in the SHRC. Shading denotes 95% prediction intervals. (**B**) Annual NPP and trend in low-altitude (red), mid-altitude (green), and high-altitude (blue) regions. β and R^2^ represent the linear regression slope and coefficient of determination, respectively. Shading denotes 95% prediction intervals. (**C**) The trend of NPP at different altitudes. The central line in the boxplot represents the median value, with box limits showing upper and lower quantiles and whiskers showing 1.5× interquartile range. Symbols a, b, and c denote significant differences (*p* < 0.05) between different altitudes as determined by the Mann-Whitney U test. The letter “a” represents the year.

At the regional scale, the NPP in the SHRC showed a significant increasing trend from 2001 to 2021, with a slope of 2.13 ± 0.78 g m^−2^ a^−1^ (Figure 4A). However, the increase of NPP decreased with altitude (Figure 4B,C) with the annual mean variability of NPP ranging from 1.52 (low-altitude) to 2.61 (high-altitude) g m^−2^ a^−1^. The trend of NPP exhibited a significant difference with altitude (Figure 4C).

**Figure 5:**
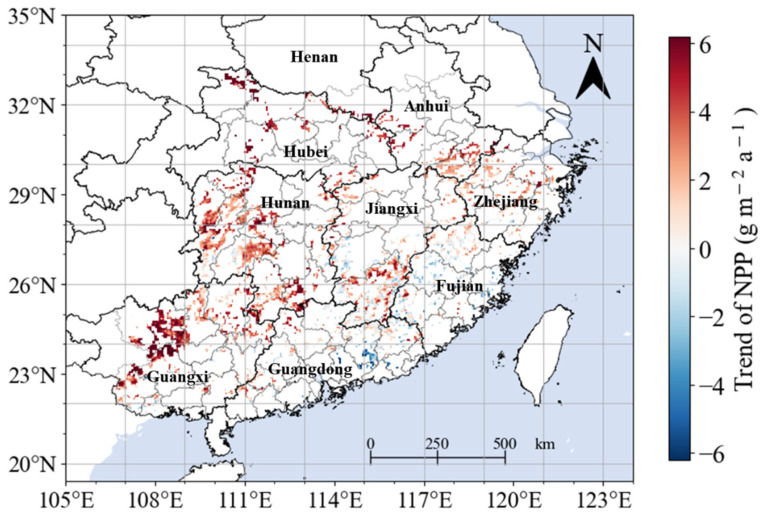
Spatial distribution of linear trends in NPP from 2001–2021. The letter “a” represents the year.

Spatially, the regions exhibiting a rising trend in annual NPP accounted for 77.9% of the overall study area. Notably, 39.5% of individual pixels displayed a statistically significant increase (*p* < 0.05), as illustrated in Figure 5. This significant increasing trend was primarily concentrated in Hubei Province, Hunan Province, Guangxi Province, the southern part of Anhui Province, the southern part of Jiangxi Province, and the central part of Zhejiang Province (Figure 5, Table 1), These findings suggest a significant ecological shift or enhancement in vegetation productivity within these specified regions (Figure 5). Conversely, there was a small number of pixels (2.5%) with statistically significant decreasing trends in NPP (*p* < 0.05). These were predominantly located in the east of Guangdong Province and in central Fujian Province (Figure 5, Table 1). At the provincial level, the proportion of pixels with a significant increasing trend far exceeded that of those with a significant decreasing trend in all the provinces except for Guangdong and Fujian (Table 1).

### 3.3. NPP in Relation to Climate Factors

At the regional scale, vegetation NPP in the SHRC exhibits a significantly positive correlation with T_mean_, with a partial correlation coefficient of 0.60 (*p* < 0.05), while its correlation with precipitation is not statistically significant (*p* > 0.05). Along the elevation gradient, after eliminating the influence of precipitation, vegetation NPP shows a significant positive correlation (*p* < 0.05) with T_mean_ in low, mid, and high-altitude hilly areas, with partial correlation coefficients of 0.58, 0.61, and 0.62, respectively. However, after removing the influence of T_mean_, the correlation between vegetation NPP and precipitation is not significant across the various altitudes.

**Figure 6:**
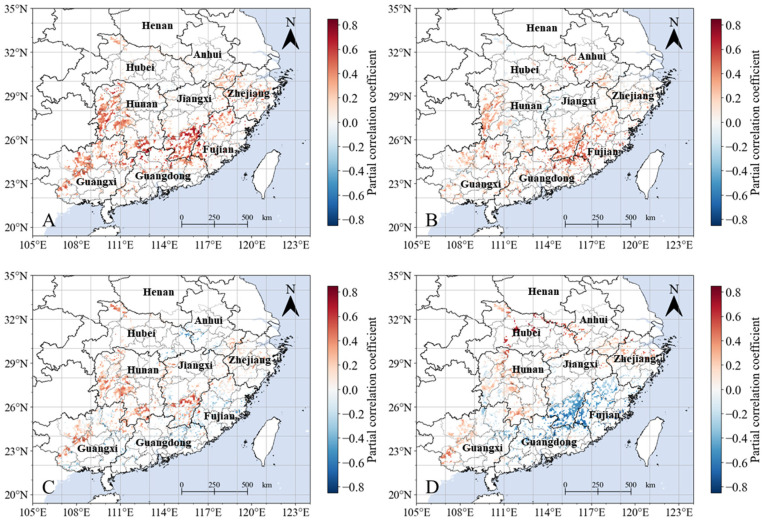
Partial correlation coefficient of annual NPP with mean daily temperature (**A**), daily maximum temperature (**B**), daily minimum temperature (**C**), and precipitation (**D**) in the southern hilly region of China from 2001–2021.

In terms of spatial patterns, after eliminating the influence of precipitation, NPP exhibits a positive correlation with T_mean_ for 90.7% of the pixels, with 21.5% of these pixels showing a significant positive correlation. These pixels are primarily located in the western part of Hunan Province, southern Jiangxi Province, northern Guangxi Province, and the western regions of Fujian Province (Figure 6, Table 2). In contrast, although 9.3% of pixels show a negative correlation between NPP and T_mean_, less than 0.1% pass the significance test (Figure 6). After removing the influence of T_min_ and precipitation, 83.0% of the pixels in the study region exhibit a positive correlation between NPP and T_max_, with only 10.3% of these, primarily located in the western part of Hunan Province, northern Guangxi Province, southern Jiangxi Province, northern Guangdong Province, and the western part of Fujian Province (Figure 6, Table 2), passing the significance test. Similarly to T_mean_, areas where NPP and T_max_ exhibit a significant negative correlation make up less than 0.1% of the study region. After removing the influence of T_max_ and precipitation, 58.2% of the pixels exhibit a positive correlation between NPP and T_max_, with only 5.0% of these passing the significance test. In this case, the pixels with a significant positive correlation are primarily located in the northern part of Guangxi Province, southern Jiangxi Province, and the western part of Hunan Province (Figure 6, Table 2). Furthermore, after removing the influence of T_mean_, 50.9% of the pixels exhibit a positive correlation between NPP and precipitation. Among these, the proportion passing the significance test is 7.0%, and these are primarily located in the western part of Guangxi Province, western Hunan Province, and central Zhejiang Province (Figure 6, Table 2). In 49.1% of the SHRC, there is a negative correlation between NPP and precipitation. Of these pixels, 16.3%—primarily located in the southern part of Jiangxi Province, northern Guangdong Province, western Fujian Province, and the eastern part of Guangxi Province (Figure 6, Table 2)—exhibit a significant negative correlation.

## 4. Discussion

Over the past 20 years, the SHRC has exhibited a significant increasing trend in vegetation NPP. This trend aligns with the increased NPP observed in land vegetation in the Northern Hemisphere [41,42] and throughout China [4,43,44] and underscores the vital role of SHRC vegetation in carbon sequestration in recent decades. However, our study reveals notable spatial heterogeneity in the overall trend of vegetation NPP. Inland areas, such as northern Guangxi, western Hunan Province, and southern Jiangxi Province, have experienced a higher rate of NPP increase, while many coastal areas in provinces such as Guangdong and Fujian show a declining trend in NPP. These results may be related to the shift in land-use types induced by economic development, as significant urban expansion has counteracted the increase in NPP caused by climate change [45]. Furthermore, our research identifies parallels with other responses to global climate change, such as mountain tree-line dynamics [10]. Interestingly, the NPP growth rate is significantly higher in low-altitude hilly areas than in high-altitude hilly areas (Figure 4) despite an overall lower NPP at low altitudes. This suggests that, in the context of global change, differences in vegetation NPP with altitude are diminishing or converging.

Previous studies on high-latitude regions [26,46], high-altitude areas [28,47,48], and arid regions [27,49] have shown significant differences in the impact of daytime and nighttime temperatures on vegetation activity. For instance, studies by Piao et al. [46] revealed that in many high-latitude regions of the Northern Hemisphere, the timing of the onset of greening for plants is primarily determined by pre-season daytime temperatures. Shen et al. [47] found that nighttime temperatures have a greater impact on vegetation greening in the Qinghai–Tibet Plateau region than daytime temperatures, while Yang et al. [49] discovered that nocturnal warming, rather than daytime warming, enhanced carbon sequestration and increased the resilience of the Inner Mongolia semi-arid temperate grassland community to drought, promoting ecosystem sustainability. Similarly, our study demonstrates that in the SHRC, vegetation responds asymmetrically to daytime and nighttime warming. Daytime warming benefits the enhancement of vegetation productivity in most areas, while the impact of nighttime warming on vegetation shows significant spatial heterogeneity (Figure 6). Although nighttime warming tends to have adverse effects on vegetation productivity in Fujian and Guangdong provinces (Table 2), it positively influences vegetation productivity in Hunan and Jiangxi provinces over much larger areas than those that are affected significantly negatively (Table 2).

This study utilized the MOD17A3HGF data product to analyze spatiotemporal variations of vegetation NPP in the SHRC from 2001 to 2021 and its response to climatic factors. Although the research results are generally consistent with previous studies in the region, demonstrating robustness [44], the study does have some limitations. Due to the large coverage and diverse vegetation types in the study area, as well as significant variations in NPP for different vegetation types under different climatic and seasonal conditions, future research should focus on further subdividing vegetation types and exploring the changing trends of vegetation NPP in different seasons and over longer time scales in the SHRC. Additionally, in analyzing the relationship between NPP and climatic factors, here we only considered multiple temperature factors and precipitation and did not incorporate other factors such as the regional atmospheric circulation, solar radiation, evapotranspiration, atmospheric water vapor or cloud cover into the impact of climate change on vegetation NPP [25,27,50]. These omissions may introduce some uncertainty into the results. Furthermore, a specific analysis of the main driving factors of human activities was not conducted. In the 21st century, substantial ecological construction initiatives have been implemented in the SHRC. These initiatives encompass projects focused on soil and water conservation, ecological restoration, species protection, vegetation recovery, and preservation, among others [51,52]. Undoubtedly, these ecological projects exert an influence on vegetation NPP. Therefore, any future research should include additional climate factors in the models and provide a more detailed analysis of human activities, such as urban expansion and ecological construction, to enhance the accuracy and reliability of results regarding the factors influencing vegetation NPP.

## 5. Conclusions

This study, spanning the period 2001 to 2021 and utilizing remote sensing and meteorological data, aims to reveal spatiotemporal patterns in vegetation productivity and climate factors in the SHRC. We explored interannual variations of vegetation productivity along the altitudinal gradient and investigated the response of NPP to various climate factors. Over the period from 2001 to 2021, we found a significant increasing trend in annual average NPP of 2.13 ± 0.78 g m^−2^ a^−1^, which exhibited variation along the altitudinal gradient. Despite an overall upward trend in NPP over various elevations, lower elevations present a faster rate of increase, indicating diminishing differences in NPP with altitude. At the regional scale, vegetation NPP exhibits a significant positive correlation with T_mean_, while the correlation with precipitation is not significant. At the pixel scale, the overall temperature rise positively influences the southern hilly region, while the impact of precipitation on NPP displays spatial heterogeneity. Regions with significant negative correlations between vegetation NPP and precipitation are concentrated in the southern areas of Guangdong, Fujian, and Jiangxi provinces. Conversely, regions further from the southeastern coast tend to exhibit positive correlations. In terms of temperature influence, the positive impact of daytime warming on vegetation NPP is more pronounced than the impact of nighttime temperature changes. 

## Figures and Tables

**Table 1:** Percentage of pixels (%) showing different trends in NPP from 2001–2021 across different provinces.

Province	Increase	Significant Increase	Decrease	Significant Decrease
Anhui	99.9	74.6	0.1	0.1
Fujian	44.2	5.7	55.8	3.5
Hubei	98.9	67.8	1.1	0.5
Hunan	93.0	64.3	7.0	0.7
Guangdong	49.8	9.7	50.2	10.3
Guangxi	85.1	45.0	14.9	0.4
Jiangxi	74.4	32.9	25.6	2.6
Zhejiang	91.4	30.3	8.6	0.4

**Table 2:** Percentages of statistically significant pixels for the partial correlation coefficients between NPP and T_mean_, precipitation, T_max_ and T_min_ for each province (%).

Province	T_mean_			Precipitation			T_max_			T_min_		
	+	−	Total	+	−	Total	+	−	Total	+	−	Total
Anhui	0.8	0.0	0.8	14.0	0.1	14.1	4.6	0.0	4.6	0.3	2.2	2.5
Fujian	25.7	0.1	25.8	0.0	57.6	57.7	28.2	0.0	28.2	1.4	7.6	9.0
Hubei	1.6	0.0	1.6	55.0	0.0	55.0	5.9	0.0	5.9	7.7	5.8	13.5
Hunan	29.1	0.0	29.1	2.8	0.7	3.5	3.6	0.0	3.6	8.7	0.1	8.8
Guangdong	23.5	0.1	23.6	0.0	33.1	33.1	20.4	0.0	20.4	0.7	3.9	4.6
Guangxi	19.0	0.0	19.0	1.9	2.8	4.7	8.5	0.0	8.5	4.3	3.6	7.9
Jiangxi	32.1	0.0	32.1	1.6	28.1	29.7	8.1	0.0	8.1	10.2	0.3	10.5
Zhejiang	12.2	0.1	12.3	7.9	1.1	9.0	0.6	0.0	0.6	1.1	0.2	1.3

Symbols “+” and “−” indicate statistically significant (*p* < 0.05) positive and negative correlation, respectively.

## Data Availability

Data will be made available on request.
